# The Phosphorylation Profile of Myosin Binding Protein-C Slow is Dynamically Regulated in Slow-Twitch Muscles in Health and Disease

**DOI:** 10.1038/srep12637

**Published:** 2015-08-19

**Authors:** Maegen A. Ackermann, Jaclyn P. Kerr, Brendan King, Christopher W. Ward, Aikaterini Kontrogianni-Konstantopoulos

**Affiliations:** 1 University of Maryland, School of Medicine, Department of Biochemistry and Molecular Biology, Baltimore, MD, USA; 2 University of Maryland, School of Medicine, Department of Physiology, Baltimore, MD, USA; 3 University of Maryland, School of Nursing, Baltimore, MD, USA

## Abstract

Myosin Binding Protein-C slow (sMyBP-C) is expressed in skeletal muscles where it plays structural and regulatory roles. The functions of sMyBP-C are modulated through alternative splicing and phosphorylation. Herein, we examined the phosphorylation profile of sMyBP-C in mouse slow-twitch soleus muscle isolated from fatigued or non-fatigued young (2-4-months old) and old (~14-months old) wild type and *mdx* mice. Our findings are two-fold. First, we identified the phosphorylation events present in individual sMyBP-C variants at different states. Secondly, we quantified the relative abundance of each phosphorylation event, and of sMyBP-C phospho-species as a function of age and dystrophy, in the presence or absence of fatigue. Our results revealed both constitutive and differential phosphorylation of sMyBP-C. Moreover, we noted a 10–40% and a 25–35% reduction in the phosphorylation levels of select sites in old wild type and young or old *mdx* soleus muscles, respectively. On the contrary, we observed a 5–10% and a 20–25% increase in the phosphorylation levels of specific sites in young fatigued wild type and *mdx* soleus muscles, respectively. Overall, our studies showed that the phosphorylation pattern of sMyBP-C is differentially regulated following reversible (i.e. fatigue) and non-reversible (i.e. age and disease) (patho)physiological stressors.

Myosin Binding Protein-C (MyBP-C) comprises a family of accessory proteins in striated muscles that contributes to the assembly and stabilization of thick filaments and modulates the formation of actomyosin cross-bridges^1^,^2^,^3^,^4^,^5^,^6^,^7^,^8^,^9^. Encoded by the *MYBPC1* gene, the slow (s) skeletal isoform of MyBP-C is composed of seven immunoglobulin (Ig) and three fibronectin-III (Fn-III) domains. At the NH_2_-terminus of the protein, upstream of the first Ig domain, C1, is a sequence of ~50 amino acids enriched with proline (Pro) and alanine (Ala) residues, referred to as the Pro/Ala rich motif. Immediately following Ig C1, is a conserved linker region of ~100 amino acids, termed the M-motif. sMyBP-C differs from the cardiac and fast skeletal homologues, as it consists of a heterogeneous family of proteins, ranging in size from ~126 to ~131.5 kDa, resulting from extensive exon shuffling^8^,^10^. Five full-length sMyBP-C variants have been identified in the mouse transcriptome, however many of the known human variants likely correspond to unidentified mouse variants. To date, fourteen sMyBP-C transcripts have been identified in the human transcriptome, encoding fourteen unique variants, which differ by small segments of amino acids within the Pro/Ala rich motif, the M-motif, Ig domain C7, and the extreme COOH-terminus^10^. The different sMyBP-C variants are co-expressed in variable amounts and combinations in both slow and fast twitch skeletal muscles, and can co-exist within a single myofiber exhibiting distinct topographies and functions^1^,^2^,^8^.

The NH_2_-terminus of sMyBP-C is phosphorylated by both PKA and PKC^11^. In particular, within the Pro/Ala rich region of the mouse (m) sequence, mSer59 and mSer62 are phosphorylated by PKA and mThr84 is phosphorylated by PKC. In addition, mSer204 within the M-motif is a substrate of both PKA and PKC. Of the four phosphorylation sites, mSer62 and mThr84 are constitutively expressed in all known mouse and human variants (Fig. 1, grey highlighted residues), while mSer59 and mSer204 are encoded by exons that are alternatively spliced and are thus present only in select variants (Fig. 1, purple and green highlighted residues, respectively). Combined, alternative splicing and phosphorylation may regulate the activities of the different variants of sMyBP-C.

Using a panel of phospho-specific antibodies in combination with conventional and phosphate-affinity gel electrophoresis, we examined the phosphorylation profile of sMyBP-C in young (2–4 months old) and old (~14 months old) wild type and *mdx* soleus muscles that were rested or subjected to an *in vitro* fatigue protocol^12^. Although the overall phosphorylation levels of sMyBP-C remain relatively unchanged between young and old wild type and *mdx* soleus muscles, we observed qualitative and quantitative differences in individual phosphorylation events or combinations thereof as a result of age, fatigue, and/or disease. Collectively, our studies are the first to demonstrate that the phosphorylation pattern of sMyBP-C is differentially regulated in response to age, fatigue, and disease in the slow-twitch soleus muscle.

## Results

### Intra- and inter-dependence of sMyBP-C phosphorylation within the Pro/Ala rich and M motifs

**Figure 1:**
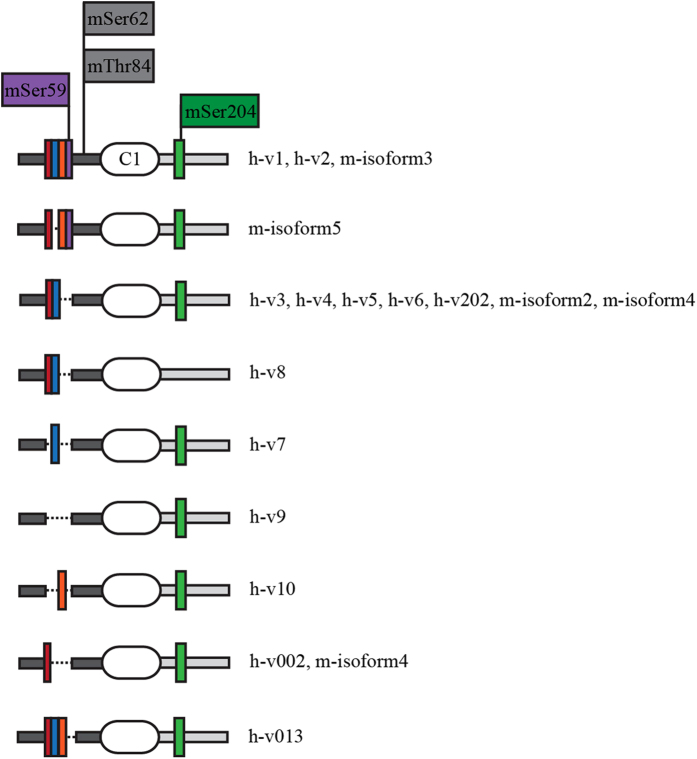
sMyBP-C is phosphorylated within its NH_2_-terminus. Schematic representation of the NH_2_-terminus of the known human and mouse variants of sMyBP-C with the phosphorylation sites highlighted. The Pro/Ala rich and M- motifs are denoted in dark and light grey, respectively. The first Ig domain (C1) is shown as a white oval. Colored rectangles indicate short stretches of amino acids that are products of alternatively spliced regions.

**Figure 2:**
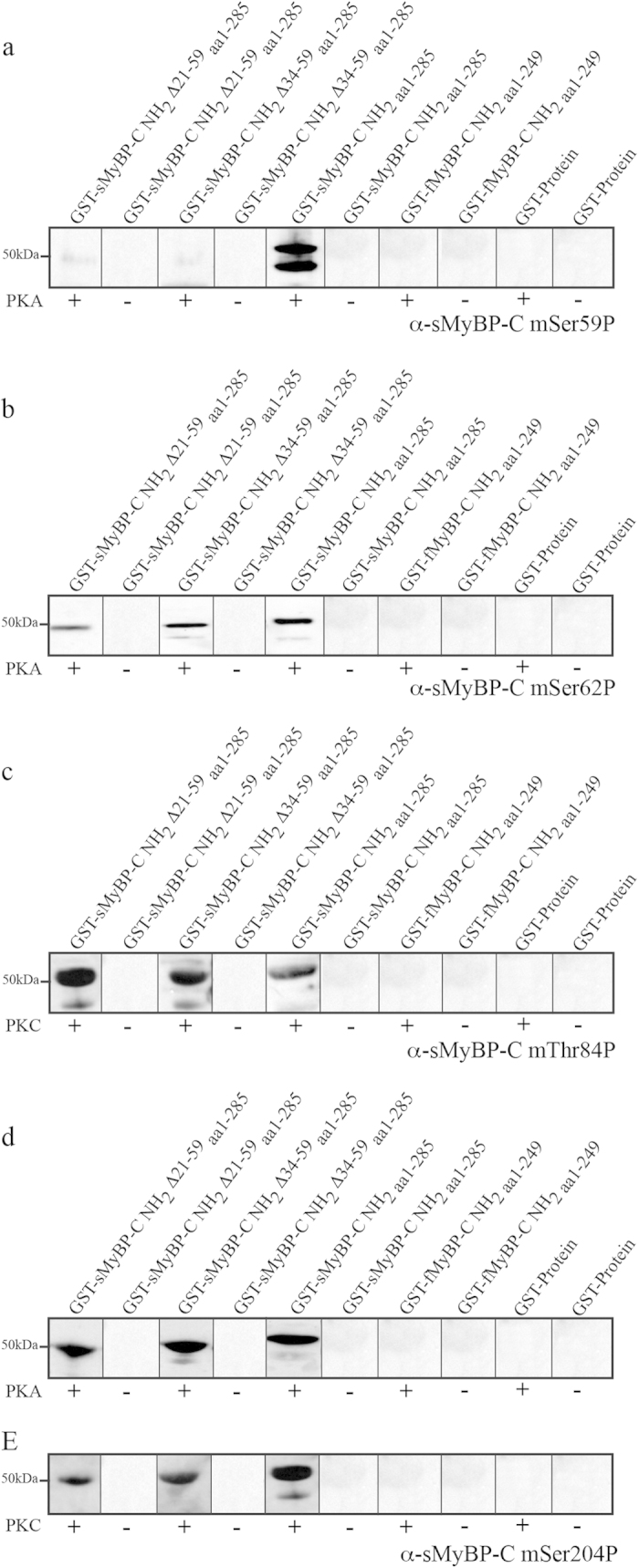
Characterization of the four α-sMyBP-C mSer59P, α-sMyBP-C mSer62P, α-sMyBP-C mThr84P and α-sMyBP-C mSer204P phospho-specific antibodies. *In vitro* kinase assays were used to determine the specificity of each phospho-antibody. The NH_2_-termini of sMyBP-C and the NH_2_-terminus of fMyBP-C were produced as GST-fusion proteins and treated with PKA and/or PKC depending on the phospho-antibody tested. Untreated and treated recombinant proteins were separated via standard SDS-PAGE and probed with the appropriate phospho-specific antibody to mSer59 (**a**) mSer62 (**b**) mThr84 (**c**) and mSer204 (**d–e**). Notably, none of the phospho-antibodies recognized the untreated NH_2_-terminus of the slow isoform or the PKA- or PKC-treated NH_2_-terminus of the fast isoform or control-GST. Therefore, these antibodies are specific for their respective phosphorylated residues present in the NH_2_-terminus of sMyBP-C.

**Figure 3:**
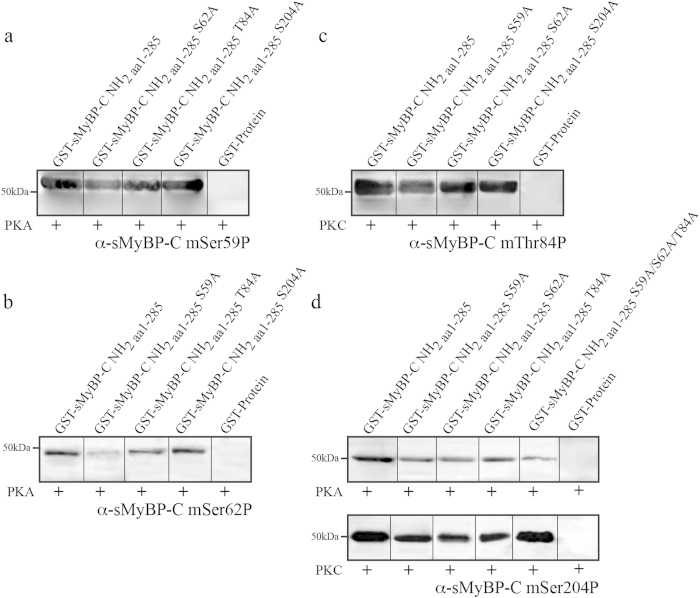
Assessment of intra- and inter-dependent phosphorylation events within the NH_2_-terminus of sMyBP-C. *In vitro* kinase assays were used to determine the intra- and inter-dependence of sMyBP-C phosphorylation events within the Pro/Ala rich motif and between the Pro/Ala rich motif and M-motif. Wild type and phospho-ablated mutant recombinant proteins containing the NH_2_-terminus of sMyBP-C corresponding to m-isoform3 were produced as GST-fusion proteins and incubated with PKA and/or PKC. Treated proteins were separated by standard SDS-PAGE, and probed with the appropriate phospho-specific antibody to mSer59 (**a**) mSer62 (**b**) mThr84 (**c**) and mSer204 (**d**).

To assess the intra-dependence of phosphorylation within the Pro/Ala rich motif of sMyBP-C (Fig. 1), we performed *in vitro* kinase assays using a series of phospho-ablated peptides along with our panel of phospho-specific antibodies (Fig. 2). In particular, we used wild type and phospho-ablated recombinant peptides corresponding to the NH_2_-terminus of m-isoform3 (GST-sMyBP-C NH_2 aa1-285_) as it contains all known phosphorylation sites, including mSer-59 and mSer-204 present only in select variants (Fig. 1). We found that phosphorylation of mSer59 is dependent upon phosphorylation of mSer62, but not of mThr84 (Fig. 3a, lane 3), given a 65% reduction in its phosphorylation levels when mSer62 is mutated to an alanine (Fig. 3a, lane 2) compared to control wild type protein (Fig. 3a, lane 1). Similarly, phosphorylation of mSer62 or mThr84 is dependent upon phosphorylation of mSer59. Accordingly, we observed a 50% and 20% reduction in the phosphorylation levels of mSer62 (Fig. 3b, lane 2) and mThr84 (Fig. 3c, lane 2), respectively, when mSer59 is mutated to an alanine. Moreover, we note a 30% reduction in the phosphorylation levels of mSer62 (Fig. 3b, lane 3) when mThr84 is mutated to an alanine. On the contrary, substitution of mSer62 by alanine failed to affect the phosphorylation levels of mThr84 (Fig. 3c, lane 3) compared to wild type control levels (Fig. 3b,c, lane 1).

We next examined the inter-dependence of phosphorylation between the Pro/Ala rich region and the M-motif. Phosphorylation of mSer204 within the M-motif via PKA is reduced by 27%, 28% and 15% when mSer59 (Fig. 3d, top panel, lane 2), mSer62 (Fig. 3d, top panel, lane 3), and mThr84 (Fig. 3d, top panel, lane 4) are mutated to alanine, respectively, compared to wild type (Fig. 3d, top panel, lane 1). Similarly, PKC phosphorylation of mSer204 is reduced by 15%, 24%, and 19% when mSer59 (Fig. 3d, bottom panel, lane 2), mSer62 (Fig. 3d, bottom panel, lane 3), and mThr84 (Fig. 3d bottom panel lane 4) are substituted by alanine, respectively, compared to wild type phosphorylation levels (Fig. 3d, bottom panel, lane 1). Moreover, PKA-mediated phosphorylation of mSer204 is highly dependent upon the simultaneous phosphorylation of the three (known) sites within the Pro/Ala rich motif, given the dramatic reduction (50%) in its phosphorylation levels when mSer59, mSer62, and mThr84 simultaneously mutated to alanine (Fig. 3d, top panel, lane 5). On the contrary, PKC-mediated phosphorylation of mSer204 is independent of the concurrent phosphorylation of the Pro/Ala rich motif sites as there is no reduction in its phosphorylation levels when mSer59, mSer62, and mThr84 are substituted by alanines (Fig. 3d, bottom panel, lane 5). Thus, PKA and PKC phosphorylation of mSer204 within the M-Motif is differentially dependent upon individual and combinatorial phosphorylation events within the Pro/Ala rich motif. Remarkably, phosphorylation of mSer59, mSer62, or mThr84 within the Pro/Ala rich motif is independent of phosphorylation of mSer204 within the M-motif (Fig. 3a–c, lane 4).

### Examination of the phosphorylation profile of sMyBP-C in the slow-twitch soleus muscle in health, fatigue, and dystrophy

To study the phosphorylation profile of sMyBP-C *in vivo*, we chose the mouse slow-twitch soleus muscle as model system due to the abundant expression of several sMyBP-C variants^8^,^10^. In particular, we generated lysates of soleus muscles obtained from: 1. young (~2 months old) and aged (~14 months old) wild type mice, 2. age-matched *mdx* mice (model of dystrophin-deficient muscular dystrophy), and 3. young (~4 months old) wild type and *mdx* mice exposed to an acute *in vitro* fatigue protocol. The lysates were analyzed by standard or phosphate-affinity SDS-PAGE immunoblotting. Given the presence of multiple sMyBP-C variants that have similar molecular weights (126–131.5 kDa)^10^, the immunoreactive bands detected in our phosphate-affinity blots may correspond to distinct variants phosphorylated to various extents, differentially phosphorylated forms of the same variant, or a combination of both. Therefore, we refrain from referring to those as hyperphosphorylated or hypophosphorylated species; we instead refer to them as low, intermediate, or high mobility species.

**Figure 4:**
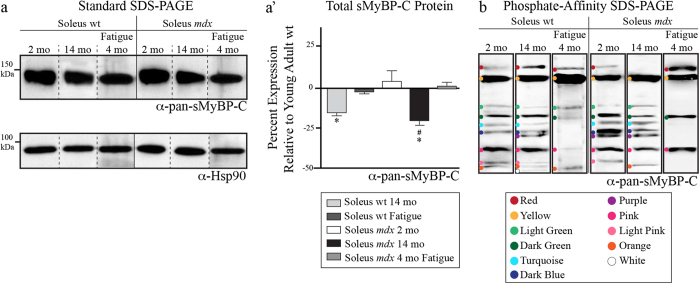
Examination of the phosphorylation profile of sMyBP-C in young and old wild type and *mdx* soleus muscles in the absence or presence of fatigue. (**a**) Western blot analysis of protein lysates separated by standard SDS-PAGE and prepared from six soleus muscle groups, including: 1. young (~2 months) wild type (n = 7), 2. old (~14 months) wild type (n = 4), 3. young (~4 months) wild type subjected to fatigue (n = 3), 4. young (~2 months) *mdx* (n = 4), 5. old (~14 months) *mdx* (n = 4), and 6. young (~4 months) *mdx* subjected to fatigue (n = 3). Samples were probed with a pan-specific antibody recognizing all variants of sMyBP-C, α-pan-sMyBP-C, and α-Hsp90 to ensure equal loading. (**a’**) Calculation of the percent (%) expression of total sMyBP-C in the different soleus muscle groups, after normalization to the expression levels of Hsp90, relative to the percent (%) expression of sMyBP-C in young wild type tissue, which was set as baseline. Significance was calculated via student’s t-test (p < 0.01). The symbols * and # denote statistical significance compared to young wild type and young *mdx* tissue, respectively. (**c**) Separation of the same protein lysates used in panel (**a**) by phosphate affinity SDS-PAGE followed by immunoprobing with the α-pan-sMyBP-C antibody. Colored dots denote immunoreactive bands of low (red and yellow dots), intermediate (light green, dark green, turquoise and dark blue dots), and high (purple, pink, light pink, orange and white dots) mobility.

We first examined the total levels of sMyBP-C by standard SDS-PAGE immunoblotting using the α-pan-sMyBP-C antibody. We found a modest, yet significant, reduction in sMyBP-C levels in aged wild type (~15%), but not in fatigued young wild type tissue, compared to un-fatigued young wild type soleus (Fig. 4a and a’). Moreover, the total levels of sMyBP-C remain unaltered in young *mdx* fatigued and un-fatigued samples, but significantly decrease (~20%) in the respective old *mdx* muscles (Fig. 4a and a’).

Evaluation of the phosphorylation status of sMyBP-C in young wild type soleus muscle using the α-pan-sMyBP-C antibody and phosphate-affinity SDS-PAGE revealed the presence of nine distinct species corresponding to differentially phosphorylated forms of the sMyBP-C variants (Fig. 4b). For ease of presentation, we denote the observed immuno-reactive bands by colored dots. We detected two low mobility bands (red and yellow dots), four intermediate mobility bands (light green, dark green, turquoise, and dark blue dots), and three high mobility bands (pink, light pink, and orange dots). Aged wild type soleus muscle expressed eleven distinct phosphorylated species sharing nine common species with young wild type samples and possessing two new intermediate (purple dot) and high (white dot) forms (Fig. 4b). In contrast, fatigue in young wild type soleus muscle significantly altered the phosphorylation pattern of sMyBP-C, as evidenced by the disappearance of three intermediate (turquoise, dark blue, and purple dots) and two high (light pink and brown dots) mobility forms (Fig. 4b).

Similar analysis of the phosphorylation profile of sMyBP-C in young and aged *mdx* soleus muscles demonstrated that they express the same major species as young wild type soleus muscle with the following exceptions: both young and old *mdx* soleus muscles contain an additional intermediate mobility species (purple dot) that is also present in aged wild type soleus muscle. However, young *mdx* soleus muscle lacks two high mobility species (orange and white dots), while aged *mdx* soleus muscle lacks one low mobility species (red dot) and one high mobility species (light pink dot) (Fig. 4b). Interestingly, the phosphorylation profile of sMyBP-C was significantly altered in young fatigued *mdx* soleus muscle compared to un-fatigued or fatigued young wild type or un-fatigued young *mdx* soleus muscles, containing only four main species (denoted with the red, yellow, dark green, and pink dots), and lacking five to seven species of intermediate and high mobility (Fig. 4b).

### Evaluation of the phosphorylation levels of mSer59, mSer62, mThr84, and mSer204 of sMyBP-C in health, fatigue, and dystrophy

Using our panel of phospho-specific antibodies (Fig. 2) and standard SDS-PAGE, we examined the phosphorylation levels of each identified phospho-site within the NH_2_-terminus of sMyBP-C in soleus muscles obtained from the six animal groups described above. As previously, we consider the young wild type soleus muscle as the basis for qualitative and quantitative comparisons.

**Figure 5:**
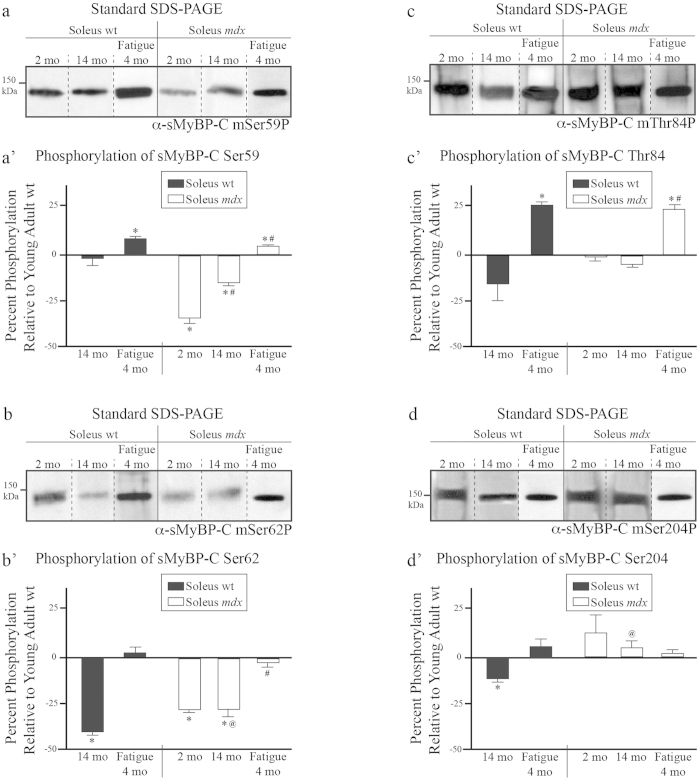
Evaluation of the phosphorylation levels of mSer59, mSer62, mThr84, and mSer204 of sMyBP-C in slow-twitch soleus muscles of young and old wild type and *mdx* animals in the presence or absence of fatigue. Western blot analyses were performed using standard SDS-PAGE and lysates prepared from the six soleus muscle groups described in Fig. 4. Samples were probed with antibodies recognizing the four known phospho-sites of sMyBP-C, including α-sMyBP-C mSer59P (**a**), α-sMyBP-C mSer62P (**b**) α-sMyBP-C mThr84P (**c**) and α-sMyBP-C mSer204P (**d**). The levels of each phosphorylated residue, mSer59 (**a’**) mSer62 (**b’**) mThr84 (**c’**) and mSer204 (**d’**) in the different soleus muscles were calculated as percent (%) expression relative to those in young wild type tissue, as described in Fig. 4. Significance was calculated via student’s t-test (p < 0.01). The symbols *, #, and @ denote statistical significance compared to young wild type, young *mdx*, and old wild type tissues, respectively.

We found that all six samples contain sMyBP-C variants that are phosphorylated at mSer59 (Fig. 5a). Notably, mSer59 is encoded by the alternatively spliced exon 5, and is only included in m-isoform3, corresponding to the NH_2_-terminus of human variants 1 and 2 (Fig. 1)^11^. The expression levels of sMyBP-C variants phosphorylated at mSer59 remain unaltered in old wild type soleus muscle; however, they modestly, yet significantly, increase in young fatigued wild type (~8%) and young fatigued *mdx* (~4%), while dramatically decrease in un-fatigued young *mdx* (~33%) and old *mdx* (~14%) soleus muscles, compared to young wild type tissue (Fig. 5a’).

Similar analysis of protein lysates with the mSer62P, mThr84P, or mSer204P antibodies indicated the presence of sMyBP-C proteins phosphorylated at the corresponding sites in soleus muscles across all conditions (Fig. 5b–d). Quantitation of the relative expression levels of phospho (p) mSer62-containing sMyBP-C proteins revealed that compared to young wild type tissue, old wild type, young *mdx,* and old *mdx* soleus muscles exhibit reduced amounts by ~39%, ~27%, and ~26%, respectively (Fig. 5b’). In contrast, the amounts of p-mSer62-containing sMyBP-C proteins remain unaltered in young fatigued wild type and *mdx* soleus muscles, compared to young untreated wild type tissue (Fig. 5b’). Similar quantitation of the relative amounts of p-mThr84-containing sMyBP-C proteins showed that they are similar between young wild type and old wild type, young *mdx* and old *mdx* soleus muscles (Fig. 5c’). However, they significantly increase in fatigued young wild type (~25%) and *mdx* (~24%) soleus muscles, compared to un-fatigued young wild type tissue (Fig. 5c’). Lastly, relative quantitation of p-mSer204-containing sMyBP-C proteins demonstrated that while fatigued (either wild type or *mdx*) and dystrophic *mdx* (either young or old) soleus muscles contain similar levels to young wild type tissue, aged wild type tissue expresses reduced (~13%) levels of such species (Fig. 5d’).

### Examination of the phosphorylation events present in individual sMyBP-C forms

**Figure 6:**
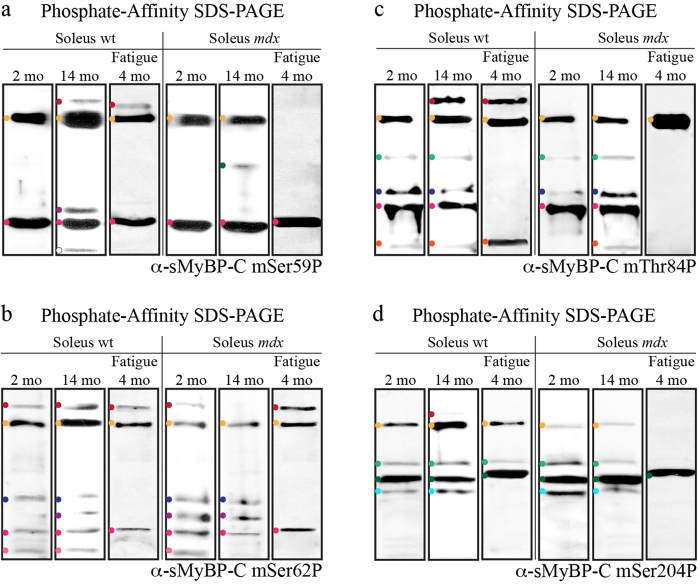
Identification of the phosphorylation events present in individual sMyBP-C forms. Western blot analysis using phosphate affinity SDS-PAGE blots of protein lysates obtained from the six soleus muscle groups described in Fig. 4 were probed with the four sMyBP-C phospho-specific antibodies: α-sMyBP-C mSer59P (**a**) α-sMyBP-C mSer62P (**b**) α-sMyBP-C mThr84P (**c**) and α-sMyBP-C mSer204P (**d**). Colored dots denote low, intermediate, and high mobility sMyBP-C phospho-species identified with each antibody, and correspond to those shown in Fig. 4.

Using phosphate affinity SDS-PAGE and our panel of phospho-specific antibodies (Fig. 2), we next examined the phosphorylation events that are present within the individual sMyBP-C phospho-species detected with our α-pan-sMyBP-C antibody (Fig. 4b). Immunoprobing of protein lysates separated via phosphate affinity SDS-PAGE with the mSer59P antibody revealed the presence of two main species, one of low mobility (yellow dot) and one of high mobility (pink dot) of equivalent abundance in young wild type soleus muscle (Fig. 6a). These are present in the soleus muscles of all groups examined, with the exception of young fatigued *mdx* tissue, which only expresses the high mobility species (Fig. 6a). Interestingly, soleus muscles from select animal groups express additional p-mSer59 sMyBP-C forms. In particular, aged wild type soleus muscle contains three additional species, including one low mobility band (red dot), one intermediate mobility band (purple dot), and one high mobility band (white dot) (Fig. 6a). The low mobility species denoted with the red dot is also detected in young fatigued wild type soleus muscle (Fig. 6a), while a species of intermediate mobility is solely observed in aged *mdx* soleus muscle (dark green dot) (Fig. 6a).

Use of the mSer62P antibody revealed a more complex profile. We observed five p-mSer62-containing species in young wild type soleus muscle (red, yellow, dark blue, pink, and light pink dots) of differential abundance. Notably, select p-mSer62-containing species exhibit similar electrophoretic mobility with p-mSer59-containing forms. The five p-mSer62-containing species are detected in protein lysates prepared from old wild type and young *mdx* soleus muscles, too, which also contain an additional intermediate mobility species (purple dot) (Fig. 6b). In contrast, fatigued young wild type or *mdx* and old *mdx* soleus muscles express only some of the five major phospho-species. In particular, fatigued young wild type or *mdx* tissues contain three major forms corresponding to two low mobility bands (red and yellow dots) and one high mobility band (pink dot) (Fig. 6b). In contrast, old *mdx* soleus muscle expresses one low mobility band (yellow dot), two intermediate mobility bands (dark blue and purple dots), and one high mobility band (pink dot) (Fig. 6b).

Similar analysis using the mThr84P antibody indicated the presence of five major p-mThr84-containing species in young wild type soleus muscle (yellow, light green, dark blue, pink, and orange dots), some of which exhibit similar electrophoretic mobility with p-mSer59- and p-mSer62-containing forms (Fig. 6c). The same pattern is observed in protein lysates prepared from aged wild type soleus muscle, which in addition expresses a lower mobility species (red dot) (Fig. 6c). Examination of the young and aged *mdx* soleus muscles indicated a similar expression profile of p-mThr84-containing species compared to young wild type soleus muscle, with the only exception being the lack of a high mobility species (orange dot) in young *mdx* tissue (Fig. 6c). Interestingly, young wild type and *mdx* soleus muscles subjected to fatigue express only two and one low mobility p-mThr84-containing species, respectively (Fig. 6c). Moreover, while the young fatigued wild type soleus muscle also contains a high mobility band (orange dot), the young fatigued *mdx* tissue lacks both intermediate and high mobility forms (Fig. 6c).

Lastly, evaluation of the expression profile of p-mSer204-containing variants indicated the presence of four main species in young wild type soleus muscle, one of low mobility (denoted with a yellow dot) and three of intermediate mobility (light green, dark green, and turquoise dots) (Fig. 6d). Examination of the number and relative abundance of p-mSer204-containing species in old wild type soleus muscle indicated the presence of the four main species detected in young wild type tissue, as well as an additional low mobility band (red dot) (Fig. 6d). Three of the four p-mSer204-containing species are also detected after exertion of fatigue in young wild type soleus muscle (Fig. 6d). Notably, the relative abundance of the intermediate mobility species denoted with a dark green dot is dramatically increased in fatigued young wild type tissue compared to untreated young wild type soleus muscle (Fig. 6d). Similar to young wild type, young and old *mdx* soleus muscles express the same four p-mSer204-containing species, while young fatigued *mdx* soleus muscle expresses only one p-mSer204-containing form of intermediate mobility (dark green dot) (Fig. 6d).

## Discussion

**Figure 7:**
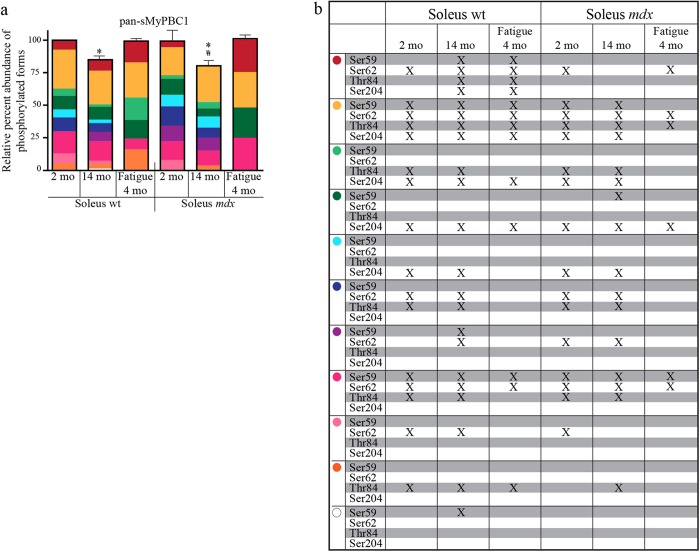
Relative abundance and phosphorylation profile of individual sMyBP-C forms. (**a**) The relative abundance of each immunoreactive band detected with the α-pan-sMyBP-C antibody following separation of protein lysates by phosphate affinity SDS-PAGE (Fig. 4B) was calculated as percent (%) expression of the total protein content within each sample. Significance is as described in Fig. 4A’. (**b**) The phosphorylation events detected in each sMyBP-C species across the six different soleus muscle groups were determined by comparative evaluation of the phosphate affinity SDS-PAGE immunoblots shown in Fig. 6. It is important to note that colored dots correspond to sMyBP-C immunoreactive bands with specific electrophoretic mobility as determined by phosphate affinity SDS-PAGE, and thus may represent different (i.e. bands denoted with red, yellow, light green, dark green, purple, and pink dots) or the same (i.e. bands denoted with dark blue, light pink, orange, and white dots) sMyBP-C phospho-species across the six soleus muscle groups.

Acute *in vitro* fatigue, aging, and chronic disease are conditions where contractility deficits have been linked to post-translational modifications of contractile proteins^13^,^14^,^15^,^16^. We used an established model of muscular dystrophy (*mdx* mouse), aging in both the *mdx* and wild type background, as well as an acute *in vitro* fatigue protocol to reveal the dynamic range of phosphorylation events in sMyBP-C in slow-twitch muscle. Herein we present evidence of the complexity of MyBP-C phosphorylation in soleus muscle. Notably, the overall abundance of sMyBP-C phosphorylation is similar across the young soleus independent of genotype or fatigue; however, both age and dystrophy result in a modest, yet significant, reduction of sMyBP-C levels (Fig. 4a). More importantly, the presence and relative abundance of individual phosphorylation events or combinations thereof vary considerably in response to age, fatigue, and/or dystrophy resulting in the expression of uniquely phosphorylated sMyBP-C species under different conditions (Fig. 7a,b). We therefore performed a comparative analysis of the data shown in Figs 3, 4, 5 to systematically present the relative abundance (Fig. 7a) and phosphorylation profile (Fig. 7B) of the individual sMyBP-C forms.

Of the nine distinct sMyBP-C forms expressed in young wild type soleus muscle (i.e. the base-line condition), a low mobility species (yellow dot, Figs 4b, 6a–d, and 7a,b) is the most abundant one (~30%), and is phosphorylated at all four known sites: mSer59, mSer62, mThr84, and mSer204. A high mobility species (pink dot, Figs 4b, 6a–d, and 7a,b) also exhibits a reasonably high abundance (~17%) and is phosphorylated at the three sites present in the Pro/Ala rich motif: mSer59, mSer62, and mThr84. The remaining species are of lower abundance (5–10%), and contain one or two (known) phosphorylation events. Interestingly, given the relative electrophoretic mobility of some of these species, it seems paradoxical that they only contain one or two phosphorylation event(s). For instance, the low mobility species marked with a red dot should represent a hyper-phosphorylated form of sMyBP-C; yet, we only detect one phosphorylation event in young wild type soleus muscle and select other samples. There are two possible explanations for this paradox: i. there are additional, still unknown, phosphorylation events; or ii. this form corresponds to a higher molecular weight variant of sMyBP-C (126–131.5 kDa) that is singly phosphorylated. Accordingly, the intermediate mobility species marked with dark green and turquoise dots that are also singly phosphorylated in some soleus samples, may correspond to higher molecular weight sMyBP-C variants, too. Conversely, the intermediate and high mobility species marked with dark blue and pink dots that contain two and three phosphorylation events, respectively, may represent lower molecular weight sMyBP-C forms.

Comparative evaluation of the phosphorylation profile of sMyBP-C across each condition revealed another peculiarity of this protein, which is reflective of its molecular complexity: immunoreactive bands exhibiting similar electrophoretic mobility among the different samples contain different numbers of phosphorylation events. For example, the low mobility band marked with a red dot is singly phosphorylated at mSer62 in young wild type and young un-fatigued and fatigued *mdx* soleus muscles, but at all four sites in young fatigued wild type, aged wild type, and all *mdx* tissues. Such differential phosphorylation patterns are observed for additional immunoreactive bands, including those denoted with yellow, light green, dark green, purple and pink dots (Fig. 7b). To reconcile this paradox, we speculate that these immunoreactive bands correspond to different sMyBP-C variants of distinct molecular weights and phosphorylation profiles. In this case, higher molecular weight variants containing one or two phosphorylation events may migrate at the same position as lower molecular weight variants containing three or four phosphorylation events. Alternatively, these immunoreactive bands may represent the same sMyBP-C variant that is differentially phosphorylated across each condition at known and/or unknown sites, but yet contains the same number of phosphorylation events.

The great majority of the identified sMyBP-C forms are phosphorylated at multiple, if not all known sites; however, phosphorylated species that contain only one (known) phosphorylation event are also present (Fig. 7b). The latter group includes sMyBP-C forms that are singly phosphorylated at any of the four identified sites, suggesting that each one of these phosphorylation events can happen independently *in vivo*. Moreover, comparative analysis of the phosphorylation profile of sMyBP-C using the α-pan-sMyBP-C and each one of the phospho-specific antibodies failed to reveal the presence of a non-phosphorylated sMyBP-C species. This observation may be interpreted in two ways. It is possible that sMyBP-C is constitutively phosphorylated at basal levels at select sites, which may change in response to different (patho)physiological stimuli, such as age, stress, fatigue or disease. Alternatively, this may be the result of non-phosphorylated higher molecular weight sMyBP-C variants co-migrating with minimally phosphorylated lower molecular weight variants.

Similar to young wild type soleus muscle, the relative abundance of individual immunoreactive bands varies significantly within each of the remaining soleus muscle groups, as well (Fig. 7a). As such, the low mobility species denoted by the red dot exhibits the highest relative abundance in young fatigued wild type and *mdx* tissues (~16–25%), while its relative expression decreases dramatically in un-fatigued wild type and *mdx* samples (~0–9%). This is not true for the other low mobility species (yellow dot), which exhibits a ~22–30% relative abundance across the different soleus muscle groups. Moreover, the intermediate mobility species (light green dot) is of low relative abundance in all muscle groups (~0–6%), with the exception of the fatigued young wild type soleus muscle (~17%). On the contrary, the intermediate mobility species (dark green dot) shows a modest and similar relative abundance (7–12%) across the different soleus groups, with the exception of young fatigued *mdx* tissue where it is significantly increased (~25%). The remaining intermediate mobility species have varied expression among the different samples, ranging between 0–11% (turquoise dot) and 0–14% (dark blue dot). Notably, both of these species are absent in young fatigued wild type and *mdx* tissues. Lastly, the five high mobility species also exhibit differential expression and abundance across the different soleus muscles. The two extremes are represented by the species marked with the pink and white dots; while the former is present in all six soleus groups with a relative abundance ranging between 8–25%, the latter is only present in aged wild type tissue where it exhibits a relatively low abundance (~2%). The other three high mobility species (purple, light pink, and orange dots) are expressed in select soleus groups and their relative expression varies between 0–13%, 0–8% and 0–16%, respectively.

Within the conditions examined, aging and dystrophy are chronic (patho)physiological states, while *in vitro* fatigue is a reversible physiological stress. We observe considerable differences in the phosphorylation profile of sMyBP-C between these reversible and non-reversible conditions; an exciting result as it likely indicates regulatory activity. Specifically, exertion of fatigue results in a significant increase in the overall phosphorylation levels of residues mSer59 and mThr84 (Fig. 5). Similarly PKC-mediated phosphorylation of titin within its PEVK region is increased following exercise^17^. This increase in phosphorylation in both titin and sMyBP-C is likely due, at least in part, to enhanced PKC enzymatic activity following acute stress^18^. On the contrary, aging and dystrophy result in a considerable reduction in the overall phosphorylation levels of residues mSer59, mSer62, and mSer204 (Fig. 5). This is consistent with the notion that PKA activity is reduced in aged soleus^19^ and dystrophic muscles, which is likely due to mislocalization of A Kinase Anchoring Proteins (AKAP), which are scaffolding proteins contributing to the proper targeting of the regulatory subunit of PKA^20^.

Our *in vitro* findings reveal intra- and inter-dependent relationships between the phosphorylation events that take place in the NH_2_-terminus of sMyBP-C. As such, individual phosphorylation events within the Pro/Ala rich motif exhibit varied dependence on the presence of additional phosphorylation events within the same motif (Fig. 3). Moreover, phosphorylation within the M-motif depends on preceding individual (in the case of PKA and PKC) or combinatorial (in the case of PKA) phosphorylation events within the Pro/Ala rich motif; however, the reverse is not true (Fig. 3).

To accurately describe the phosphorylation profile of each one of the fourteen human or five mouse slow variants is an immense task that will require a combinat.ion of highly sophisticated molecular, biochemical and proteomic approaches. These would include large-scale separation techniques, such as high-resolution two-dimensional gel electrophoresis alongside with variant-specific antibodies and/or liquid chromatography combined with advanced high-sensitivity mass spectrometry and computational methods^21^,^22^. More importantly, such approaches need to be performed at the single fiber or thick filament level under diverse conditions ranging from normalcy to exertion of different stressors, such as aging, exercise, atrophy, fatigue, injury, and disease. Nevertheless, our studies are the first to indicate that the phosphorylation pattern of the sMyBP-C sub-family is differentially regulated following (patho)physiological reversible (i.e. fatigue) and non-reversible (i.e. aging and disease) stressors. Together our results provide a foundational understanding of the phosphorylation profile and regulation of sMyBP-C, and suggest a complexity of signaling that is modified by aging, disease and acute physiological stress. With this foundation, we and others can begin to design *in vivo* and *in vitro* experiments with new molecular, biochemical and genetic tools to unravel the phosphorylation *vs.* function relationship of sMyBP-C in slow-twitch skeletal muscles.

## Methods

### Generation of Wild Type and Mutant Recombinant Proteins

Recombinant protein corresponding to the NH_2_-terminus of the mouse isoform-3 sMyBP-C sequence (sMyPB-C NH_2 aa 1-285_) was produced as a GST-fusion protein as described in^11^. Phospho-ablated mutants were created by individually or combinatorially replacing the phosphorylatable residues within the Pro/Ala rich motif (mSer-59, mSer-62, mThr-84) and the M-motif (mSer-204) with Ala using the Quickchange site-directed mutagenesis kit (Stratagene, La Jolla, CA) as previously reported^23^. Sense and antisense oligonucleotide sets are listed in Table 1. The authenticity of all constructs was verified by sequence analysis, and mutant fragments were expressed as GST-fusion proteins, as described^11^.

### *In vitro* Kinase Assays

*In vitro* kinase assays were performed as described in^11^,^24^ with minor modifications. Specifically, 150 ng/μl of purified recombinant proteins were incubated with PKA or PKC at 1 U kinase/μg protein. The assay was performed in Buffer A containing 20 mM Hepes, 100 mM KCl, 10 mM MgCl_2_, 2 mM ATP and 1 mM DTT, and the reaction mixture was incubated at 20 °C for 25 min. The assay was terminated by addition of SDS sample buffer and separated by standard SDS-PAGE, transferred to nitrocellulose, and probed with each of the phospho-specific antibodies (listed below).

### Antibodies

Mouse antibodies to pan-sMyBP-C (300 ng/ml, Abnova, Walnut, CA) and Hsp90 (300 ng/ml, Cell Signaling Technology, Inc., Danvers MA), which served as loading control, were used according to the manufacturers’ instructions. In addition, custom polyclonal antibodies to each of the four previously identified phospho-sites of sMyBP-C (mSer59, mSer62, mThr84, and mSer204;^11^), were generated. Peptides 5′ RALERKDSEW 3′, 5′ EWSLGESPAGC 3′, 5′ CANSQLSTLFVEK 3′, and 5′ RSAFKRSGEGQED 3′ containing phosphorylated mSer59, mSer62, mThr84, and mSer204 (underlined) were used to immunize rabbits for production of polyclonal antibodies (Open Biosystems, Huntsville, AL). Phospho-specific antibodies were obtained through two rounds of affinity purification, following the manufacturer’s protocols. Briefly, in the first step, immune serum was purified against the phosphorylated form of the respective antigenic peptide coupled to sepharose beads. In the second step, the eluted antibodies were further purified against the non-phosphorylated form of the respective peptide, also coupled to sepharose beads. The antibodies contained in the flow-through fraction from the second affinity column were used as affinity purified phospho-specific antibodies at 750 ng/ml in subsequent immunoblotting experiments.

### Antibody Validation and Specificity

The specificity of each custom phospho-specific antibody was verified in immunodepletion experiments, as previously reported^25^, and in *in vitro* kinase assays, as described above and in^11^,^24^. For the *in vitro* kinase assays, recombinant proteins corresponding to the NH_2_-termini of the mouse sMyBP-C sequence were produced as GST-fusion proteins^11^. The NH_2_-terminus of the fast (f) skeletal isoform of MyBP-C, fMyBP-C, including the Pro/Ala rich motif, Ig C1, and the M-motif (aa 1–249; Accession Number: NP_666301), was also produced as a GST-fusion protein and tested to ensure the specificity of the generated phospho-antibodies. Following affinity purification, the recombinant slow and fast MyBP-C NH_2_-terminal proteins and control GST-protein were either left untreated or treated with PKA and/or PKC, separated by SDS-PAGE, transferred to nitrocellulose, and probed with each of the phospho-specific antibodies (Fig. 2). As expected, the α-sMyBP-C mSer59P antibody only recognized the recombinant protein corresponding to sMyBP-C NH_2 aa1-285_, which includes the sequence encoded by exon 5 containing mSer-59; the other two sMyBP-C constructs lack exon 5.

### Animal Models and Tissue Collection

All animal procedures were performed in accordance with protocols approved by the Institutional Animal Care and Use Committee of the University of Maryland, School of Medicine. Freshly isolated soleus muscles were collected from young (~2 months) and old (~14 months) adult male wild type C57BL/6Scsn/J and dystrophic *mdx* C57BL/10Scsn-Dmd mdx/J mice (Jackson Laboratories, Bar Harbor, ME), and flash frozen in liquid nitrogen. For each experiment, four to seven (n = 4–7) different soleus muscles were used per animal group.

### Fatigue Protocol

Fatigue of the soleus muscle from young (~4 months) wild type and *mdx* mice was performed using *in vitro* methods, as previously described^12^,^26^. After the experimental protocol, the muscle was allowed to rest for 5 minutes and was then trimmed proximal to the suture connections, blotted, and flash-frozen in liquid nitrogen. The contralateral soleus muscle was surgically excised and immediately flash-frozen to serve as un-fatigued control sample. For each group of wild type and *mdx* animals, three (n = 3) soleus muscle pairs (each pair included the fatigued and contralateral un-fatigued muscle) were used.

### Generation of Protein Lysates and Western Blotting

Lysates from mouse soleus muscles were prepared as previously reported^1^. Approximately 30 μg of protein lysates from each tissue were prepared for electrophoresis and heated at 90 °C for 5 minutes. Separation of protein lysates was performed either using standard SDS-PAGE (Life Technologies, Carlsbad, CA), as reported^10^,^11^, or phosphate affinity SDS-PAGE. Evaluation of the entire lanes following immunoprobing with the pan-sMyBP-C antibody indicated the absence of any (detectable) degradation of sMyBP-C in the muscle lysates (SFig. 1). Separation via phosphate affinity SDS-PAGE was carried out according to the manufacturer’s instructions, using 100 μmol/L Phos-tag^TM^ (Wako Chemicals USA, Inc. Richmond, VA) and a 10% w/v solution of acrylamide^27^. Following electrophoretic separation, lysates were subjected to western blot analysis and probed with the indicated antibodies.

### Statistical Analysis

Quantification of the relative content of total sMyBP-C and of the relative expression of each phosphorylated residue among and within samples, respectively, was performed in standard SDS-PAGE blots by ImageJ software (NIH, Bethesda, MD). Percent (%) expression was normalized to the levels of Hsp90, which served as loading control, and subsequently to the levels of sMyBP-C present in the young wild type un-fatigued sample, which served as the basis for all our quantifications. Experiments were repeated three times, using three different muscle samples. Significance was calculated via student’s t-test (p < 0.01). In addition, we used Image J software to quantify the relative abundance of specific immuno-reactive bands within a sample. To this end, we used the quantified relative content of total sMyBP-C for each sample as a representation of total sMyBP-C protein within that sample. The relative abundance of each immuno-reactive band recognized by the pan-sMyBP-C antibody in the phosphate affinity SDS-PAGE blots was calculated as a percentage of the total protein within that sample. Lastly, we used the phosphate affinity SDS-PAGE western blots probed with each of the phospho-specific antibodies to determine the residues that are phosphorylated within a specific immuno-reactive band detected with the pan-sMyBP-C antibody.

## Additional Information

**How to cite this article**: Ackermann, M. A. *et al.* The Phosphorylation Profile of Myosin Binding Protein-C Slow is Dynamically Regulated in Slow-Twitch Muscles in Health and Disease. *Sci. Rep.* **5**, 12637; doi: 10.1038/srep12637 (2015).

## Supplementary Material

Supplementary Information

## Figures and Tables

**Table 1:** Oligonucleotides used for site directed mutagenesis.

**Mutagenesis Primers**			
**Mutation**	**Template**	**Sense Primer**	**Antisense Primer**
sMyPB-C NH_2 aa 1-285_ S59A	sMyPB-C NH_2 aa 1-285_	ATGGAGAGAAAAGATGCAGAATGGTCTCTTGGTGAG	CTCACCAAGAGACCATTCTGCATCTTTTCTCTCCAG
sMyPB-C NH_2 aa 1-285_ S62A	sMyPB-C NH_2 aa 1-285_	GAAAAGATTCAGAATGGGCTCTTGGTGAGTCACCTGCTG	CAGCAGGTGACTCACCAAGAGCCCATTCTGAATCTTTTC
sMyPB-C NH_2 aa 1-285_ T84A	sMyPB-C NH_2 aa 1-285_	CAACTCCCAGCTGTCCGCCCTGTTTGTTGAAAAACCTC	GAGGTTTTTCAACAAACAGGGCGGACAGCTGGGAGTTG
sMyPB-C NH_2 aa 1-285_ S204A	sMyPB-C NH_2 aa 1-285_	CAGATCTGCCTTCAAAAGAGCTGGAGAAGGTCAAGAGGATGC	GCATCCTCTTGACCTTCTCCATCTCTTTTGAAGGCAGATCTG
sMyPB-C NH_2 aa 1-285_ S59A/S62A	sMyPB-C NH_2 aa 1-285_ S59A	GAAAAAGATGCAGAATGGGCTCTTGGTGAGTCACCTGCTG	CAGCAGGTGACTCACCAAGAGCCCATTCTGCATCTTTTC
sMyPB-C NH_2 aa 1-285_ S59A/S62A/T84A	sMyPB-C NH_2 aa 1-285_ S59A/S62A	CAACTCCCAGCTGTCCGCCCTGTTTGTTGAAAAACCTC	GAGGTTTTTCAACAAACAGGGCGGACAGCTGGGAGTTG
