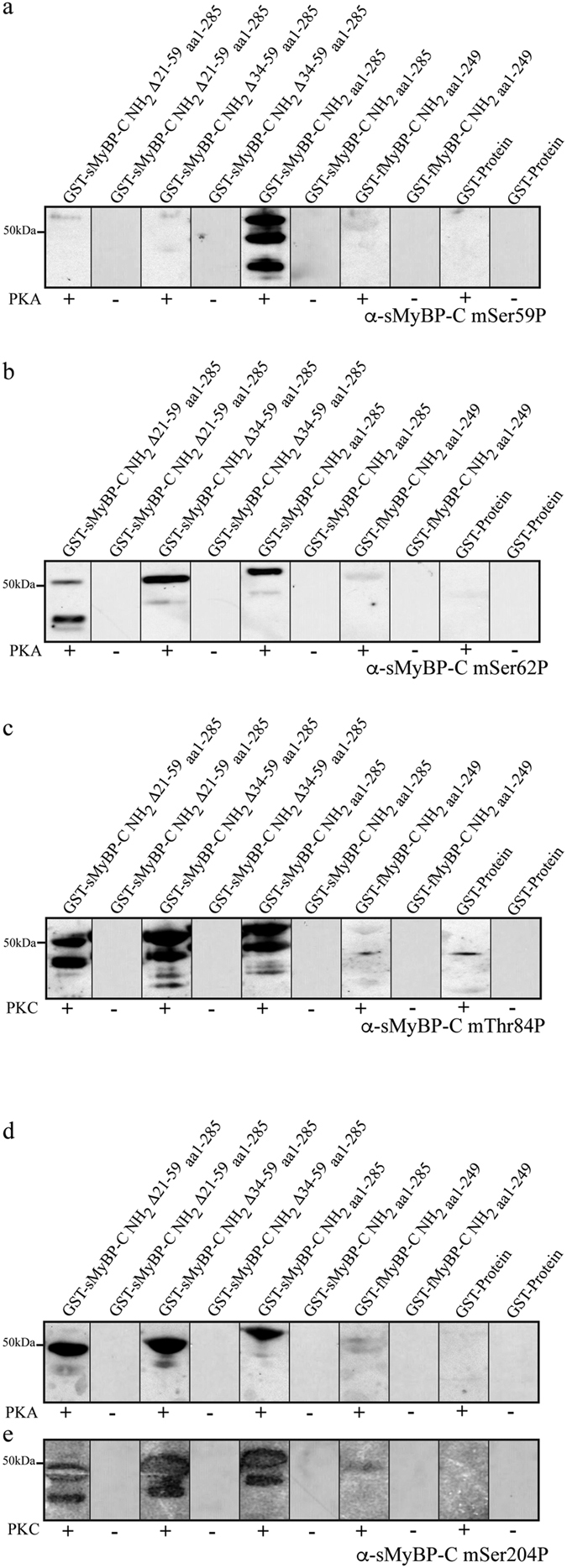# Corrigendum: The Phosphorylation Profile of Myosin Binding Protein-C Slow is Dynamically Regulated in Slow-Twitch Muscles in Health and Disease

**DOI:** 10.1038/srep46969

**Published:** 2018-05-11

**Authors:** Maegen A. Ackermann, Jaclyn P. Kerr, Brendan King, Christopher W. Ward, Aikaterini Kontrogianni-Konstantopoulos

Scientific Reports 5: Article number: 1263710.1038/srep12637; published online: 08 19 2015; updated: 05 11 2018

This Article contains errors in Figure 2, where the blots for (−) GST-sMyBP-C NH_2 aa1-285_, (+) GST-fMyBP-C NH_2 aa1-249_, (−) GST-fMyBP-C NH_2 aa1-249_, (+) GST-protein and (−) GST-protein (the right half of the panel) were duplicated between all panels. Additionally, blots for (+) GST-sMyBP-C NH_2_ Δ21-59 _aa1-285_ in panel 2c and (+) GST-sMyBP-C NH_2 aa1-285_ in panel 2e were duplicated.

The authors re-scanned the images for the blots presented in this figure. Data in panel 2a, samples (+) GST-sMyBP-C NH_2_ Δ21-59 _aa1-285_, (+) GST-sMyBP-C NH_2_ Δ34-59 _aa1-285_, (+) GST-sMyBP-C NH_2 aa1-285_, (+) GST-fMyBP-C NH_2 aa1-249,_ panel 2b, samples (+) GST-sMyBP-C NH_2_ Δ21-59 _aa1-285_, (+) GST-sMyBP-C NH_2_ Δ34-59 _aa1-285_, and (+) GST-sMyBP-C NH_2 aa1-285_, and panel 2d sample (+) GST-sMyBP-C NH_2_ Δ34-59 _aa1-285_ remain unchanged. All other images were replaced with re-scanned blots. All samples in the corrected figure are shown with the wider field of view.

The correct Figure 2 appears below as Figure 1 and the Supplementary Information file has now been replaced. In the revised version of the Supplement the unprocessed images of full-length blots for all blots presented in Figure 2 are now included as Supplementary Figures 2-6.

The conclusions of the Article remain unchanged. The authors apologize for the errors.

## Figures and Tables

**Figure 1:**