# Supplementary material for: The Phosphorylation Profile of Myosin Binding Protein-C Slow is Dynamically Regulated in Slow-Twitch Muscles in Health and Disease
Source: Sci Rep. 2015 Aug 19;5:12637. doi: 10.1038/srep12637 (PMC4642540; doi:10.1038/srep12637)
Supplement: Supplementary Information [file srep12637-s1.doc]

**The Phosphorylation Profile of Myosin Binding Protein-C Slow is Dynamically Regulated in Slow-Twitch Muscles in Health and Disease**

Maegen A. Ackermann^1^, Jackie Kerr^2#^, Brendan King^1#^, Chris Ward^3^, and

Aikaterini Kontrogianni-Konstantopoulos^1*^

*^1^University of Maryland, School of Medicine, Department of Biochemistry and Molecular Biology, Baltimore, MD, USA*

*^2^University of Maryland, School of Medicine, Department of Physiology, Baltimore, MD, USA*

*^3^University of Maryland, School of Nursing, Baltimore, MD, USA*

^#^These authors contributed equally.

Running title: MyBP-C slow phosphorylation in slow skeletal muscle

*To whom correspondence should be addressed: Aikaterini Kontrogianni-Konstantopoulos, Department of Biochemistry and Molecular Biology, University of Maryland, School of Medicine, 108 N. Greene Street, Baltimore, MD 21201, Tel: 410-706-5788, Fax: 410-706-8297, E-mail: [akons001@umaryland.edu](mailto:akons001@umaryland.edu)

**Keywords:** MYBPC1, slow-twitch skeletal muscle, phosphorylation, aging, fatigue, dystrophy, phosphate-affinity SDS-PAGE


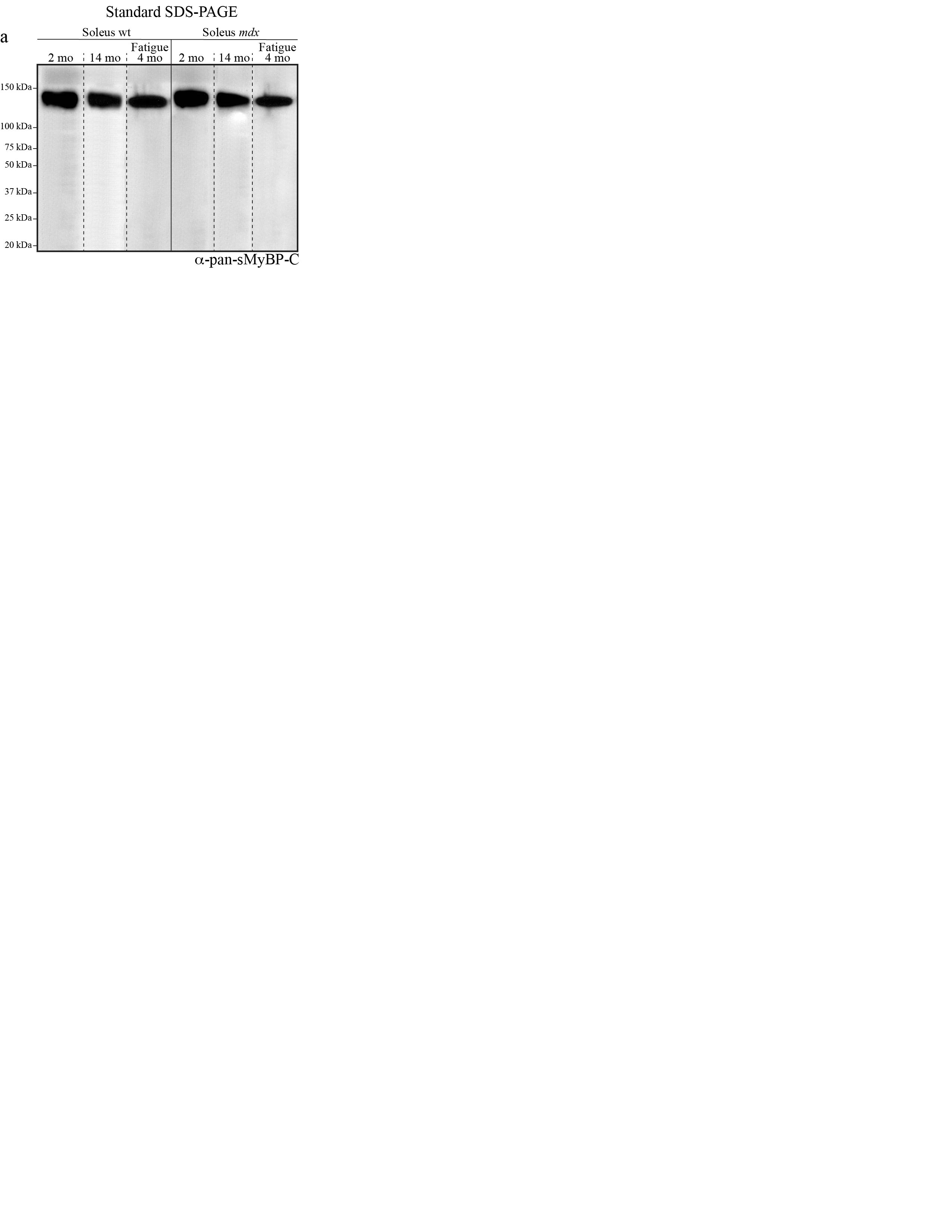
**SFIGURE 1. Evaluation of the presence of degradation in sMyBP-C in mouse soleus muscle lysates.** (A) Western blot analysis of protein lysates separated by standard SDS-PAGE, and prepared from the six soleus muscle groups described in Fig. 4. Samples were probed with a pan-specific antibody, α-pan-sMyBP-C, recognizing all variants of sMyBP-C (126-131.5 kDa). The entire lane for each sample is shown to indicate the absence of smaller immunoreactive bands, and confirm the lack of degradation of sMyBP-C.
